# Microscopic Features of Fractured Fragment of Nickel-Titanium Endodontic Instruments by Two Different Modes of Torsional Loading

**DOI:** 10.1155/2018/9467059

**Published:** 2018-02-21

**Authors:** Ibrahim H. Abu-Tahun, Sang Won Kwak, Jung-Hong Ha, Hyeon-Cheol Kim

**Affiliations:** ^1^Department of Conservative Dentistry, School of Dentistry, The University of Jordan, Amman, Jordan; ^2^Department of Conservative Dentistry, School of Dentistry, Dental Research Institute, Pusan National University, Yangsan, Republic of Korea; ^3^Department of Conservative Dentistry, School of Dentistry, Kyungpook National University, Daegu, Republic of Korea

## Abstract

This study compared the microscopic features of the fractured endodontic nickel-titanium (NiTi) rotary instruments by two different torsional loadings: repetitive torsional loading (RTL) and single torsional loading (STL) based on the International Organization for Standardization (ISO). ProTaper Next, HyFlex EDM, and V-Taper 2 were compared in this study. In the STL method, the torsional load was applied after fixing the 3 mm tip of the file, by continuous clockwise rotation (2 rpm) until fracture. In the RTL method, a preset rotational loading (0.5 N·cm) was applied and the clockwise loading to the preset torque and counterclockwise unloading to original position were repeated at 50 rpm until the file fractured. Fractured fragments by two methods were compared under a scanning electron microscope (SEM) to examine the topographic features of the fractured surfaces and longitudinal aspects. SEM examinations showed significantly different features according to the loading methods. Specimens from the RTL method showed ruptured aspects on cross sections, with multiple areas of initiated cracks while the STL method showed the typical features of torsional failure, such as circular abrasion marks and fatigue dimples. This study suggested a new repetitive torsional loading method which is much more clinically relevant and may result in a different fracture feature from STL method.

## 1. Introduction

Root canal shaping using nickel-titanium (NiTi) rotary instruments not only is easier and faster than preparation with stainless-steel (SS) manual instruments, but also causes fewer iatrogenic alterations to the original root canal shape [1]. In spite of the enhanced flexibility and strength compared with manual endodontic instruments, NiTi endodontic instruments are still subject to be broken during the clinical use [1, 2].

Literatures have reported that NiTi rotary instruments have two basic mechanisms of fracture [2–4]. Cyclic fatigue fracture or flexural failure may occur when a file rotates in a curved root canal and is caused by repeated tensile and compressive stresses [2, 4]. Meanwhile, torsional fracture is related to a continuous rotation of the engine when the instrument binds in the root canal [2].

Cyclic fatigue resistance tests for the NiTi rotary instruments have been executed extensively. On the other hand, there is little information or test protocol available for the torsional fracture resistance tests. The common method to measure the torsional resistance was conducted under a static rotational condition that is mainly based on International Organization for Standardization (ISO) 3630-1 [5]. However, its original purpose was for testing SS manual instruments. According to the ISO specification, the 3 mm file tip was fixed with the brass and 2 rpm of rotational speed was applied to generate a torsional load. However, the test conditions to create torsional load by single rotation until fracture happens do not commonly happen in clinical situation.

During the clinical use of rotary NiTi instruments, the files rotate at speeds much faster than 2 rpm with autoreverse mode and the files undergo repetitive locking and unlocking by the autoreverse motion at a prefixed level of torque. Thus, repeated locking and release of the rotary instruments by the torque-controlled motor are common in clinic [6]. Particularly, rotary instruments are subject to higher torsional stresses in narrow canals than in the wider canals; hence the chance of experiencing such repetitive torsional loads is increased [7].

The different modes of stress loading may result in different modes of mechanical failure and produce the different topographic features [2, 8]. Therefore, the torsional resistance and the fracture modes of various NiTi instruments were compared using the dynamic repetitive torsional loading method incorporating the autoreverse motion and their fractured specimens were compared with the specimens from single continuous rotation test method.

## 2. Materials and Methods

Three NiTi instrument systems made of different NiTi alloy and geometries were compared for the torsional resistance. V-Taper 2 (VTP, SS White, Lakewood, CA, USA) made of conventional NiTi alloy, HyFlex EDM (HDM, Coltene/Whaledent, Altstätten, Switzerland) produced via electrodischarge machining (EDM) using CM-wire, and ProTaper Next (PTN, Dentsply Sirona, Ballaigues, Switzerland) made of M-wire were compared using the following two torsional resistance tests. All files used in this study had the same ISO tip size of #25 and 25 mm length but with different shaft taper (0.08 taper for VT2 and variable taper for HDM and PTN). No defects and/or deformities were detected on any instrument under a dental operating microscope (Zeiss Pico, Carl Zeiss MediTec, Dublin, CA) prior to the experiments.

### 2.1. Test I: Torsional Resistance Test Using Repetitive Torsional Load (RTL)

The apical 3 mm portion of each instrument (*n* = 15 per brand) was secured between brass plates. The file was driven at 50 rpm clockwise until it achieved the preset torque of 0.5 N·cm; then it was returned to its original position while keeping the files straight using a custom-made device (DMJ system, Busan, Korea). It was considered one torsional loading cycle. The loading was repeated automatically until the file was broken. Software program operating the custom-made device automatically recorded the number of repetitive load cycles until fracture (NRCF) of each instrument.

### 2.2. Test II: Torsional Resistance Test Using Single Continuous Torsional Load (STL) Based on the ISO Specification

The apical 3 mm tip of each file was secured in the same way as Test I. The ultimate torsional strength (N·cm) until fracture was determined for each instrument (*n* = 15 per brand) using the same device, but the files were rotated at a constant rate of 2 rpm in a clockwise direction until fracture, while keeping the files straight. The ultimate torsional strength (N·cm) was recorded during the rotation of the files at the rate of 20 Hz.

### 2.3. Scanning Electron Microscope Examination

Seven fractured fragments of each instrument system from each method were randomly selected and ultrasonically cleaned with absolute alcohol for approximately 120 s. The fractured surfaces and longitudinal aspects of the fractured instruments were examined under a scanning electron microscope (SEM) (SU8220; Hitachi High Technologies, Tokyo, Japan) for the topographic features.

### 2.4. Statistic Comparison of the Data from Two Test Methods

The data were first examined using the Kolmogorov-Smirnov test for normality of distribution. The results were statistically analysed using a one-way analysis of variance (ANOVA) and Duncan post hoc comparison to identify differences between the groups. The results from the two test methods were also statistically compared for any differences between methods using the Spearman correlation test (SPSS v. 23.0 for Mac; IBM Corp, Somers, NY). All statistical analyses were completed at a significance level of 95%.

## 3. Results

The RTL test showed that the VTP group had the highest NRCF among the test groups, while the PTN group had the least NRCF (*p* < 0.05). The order of the NRCF according to the Wilcoxon rank-sum test was as follows from highest to lowest: VTP, EDM, and PTN (*p* < 0.05). The results of STL test using single continuous torsional loading showed that the VTP group also had the highest ultimate torsional strength, while the PTN group had the least strength (*p* < 0.05). When the same instrument systems underwent RTL and STL methods, the results between the two test methods were strongly correlated (correlation coefficient = 1). The RTL method yielded the same order of resistance as the STL method (Table 1).

**Figure 1:**
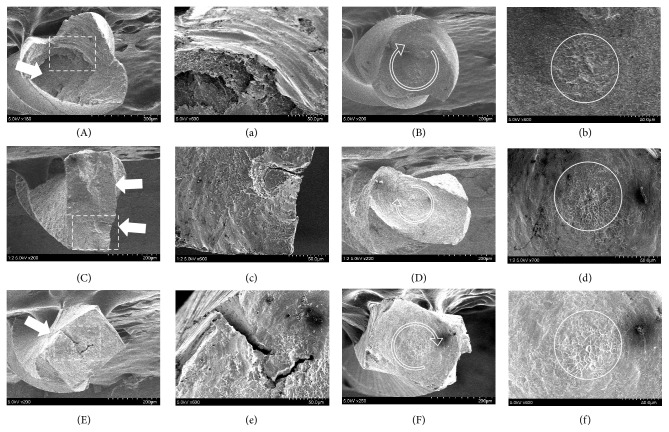
Cross-sectional aspects of representative fractured fragments from the RTL method (two left columns) and STL method (two right columns) (A, a, B, and b: V-Taper 2; C, c, D, and d: HyFlex EDM; E, e, F, and f: ProTaper Next). Specimens from the RTL method show ruptured aspects with multiple levels of fracture surfaces and gross catastrophic features (white arrows in A, C, and E) without any circular abrasion marks. Magnified dotted box (a, c, and e) shows atypical features which were not seen in two right columns of STL method. The specimens from STL method show typical features of torsional failure, such as a circular abrasion mark (arrow circle) and fatigue dimple at the center of rotation (white circle). RTL: repetitive torsional loading; STL: single continuous torsional loading.

The SEM examination of the fractured specimens from the two test methods (RTL and STL) showed significantly different features. While the examination on the cross sections of the specimens from STL method showed typical features of torsional failure, such as circular abrasion marks and fatigue dimples at the center of rotation, the RTL method showed ruptured aspects with multiple levels of fracture surfaces and gross catastrophic features without any circular abrasion marks (Figure 1). There were no specific differences among the file brands.

**Figure 2:**
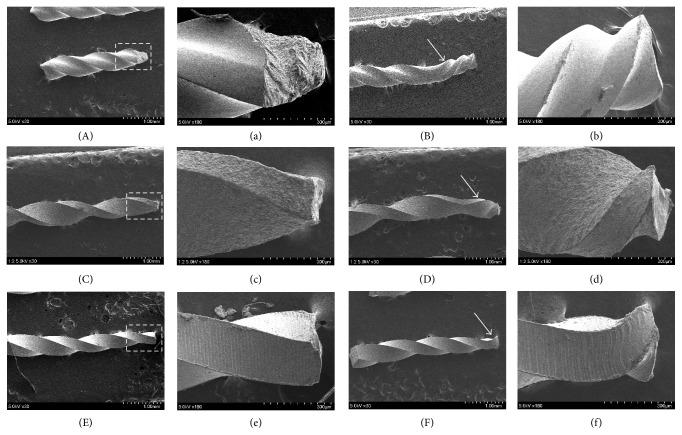
Longitudinal and lateral aspects of representative fractured fragments from the RTL method (left two columns) and STL method (right two columns). Specimens from the RTL method show rare unwinding near the fracture area, while the specimens from UTL method show typical features of unwinding (white arrow). The specimens from the RTL method show irregular and catastrophic aspects with some cracks without unwinding. RTL: repetitive torsional loading; STL: single continuous torsional loading.

On the longitudinal and lateral aspects of the fractured fragments, specimens from the RTL method showed rare unwinding near the fracture area, while the specimens from STL method had typical features of unwinding area. The specimens from the RTL method showed irregular fracture aspects with some cracks and without unwinding (Figure 2).

## 4. Discussion

By using the NiTi instruments, clinicians can predictably shape curved, well-centered root canals, with a decreased risk of transportation, ledging, and perforation compared to SS instruments [1]. Nevertheless, there still remains the risk of instrument failure with NiTi rotary instruments. Fractures of NiTi files are known to result from cyclic fatigue, torsional failure, or a combination of both [2].

While cyclic fatigue resistance is usually compared using the NCF value measured under various conditions of the radius of the canal curvature and arc length using simulated canals [2], the torsional strength of NiTi instruments has been compared mainly by the method described in the ISO 3630-1, ADA/ANSI specifications number 28 [9], or their modifications [3, 10]. However, torsional loading under such “monotonic” conditions rarely occurs clinically [11]. On the other hand, the autoreverse motion by the endodontic motor may create repetitive loading and unloading due to a certain condition from the root canal or operator. Therefore, in the present study, the increased speed of 50 rpm and the torsional loading from the repetitive methods were applied to improve the clinical relevance.

Both test methods yielded same results regarding their torsional resistance among the groups. The instruments with a relatively higher torsional resistance during the RTL method had also higher resistance with the STL method; VTP showed the highest resistance from both methods. Both methods resulted in the same order of resistance among the 3 tested groups. It means that the more clinically relevant way in RTL method has similar results to the ISO standard method and RTL method is more appropriate for the NiTi engine-driven instruments.

In the present study, VTP showed the highest torsional resistance from both methods and it might result from the reason that the VTP had bigger taper than other files compared. The HDM made of CM-wire showed higher torsional resistance than the PTN made of M-wire. Basically, the characteristics of the instruments made of CM-wire are a higher flexibility and lower stiffness, which contributed to the lower torsional resistance and strength than conventional NiTi alloy [12, 13]. However, the higher resistance of HDM made of CM-wire may result from their geometric differences, including the cross-sectional area and the special manufacturing method of EDM [4, 8, 13, 14].

Meanwhile, depending on the alloy and/or geometric differences, the file may have different mechanical properties by the repetitive loading. NiTi material of endodontic instruments is sensitive to not only thermomechanical treatment, but also repetitive mechanical stimulation (Cheung et al., 2013 [15]). Repeated loading without causing surface defects or microcracking, acting as stress raisers and being precursors of material failure, would serve to increase the density of dislocations inside the material [16, 17]. This situation could result in a higher degree of torsional resistance [15]. Since repetitive loading during the initial stage of root canal preparation in a clinical situation could change the properties and mechanical resistance of NiTi rotary instrument, the repetitive loading method could have a higher clinical relevance than the single continuous loading test. Considering the possibility of increased strength by the repeated loading, a change in the assessment method would be reasonable.

In fractographic examination, features of the specimens observed using the RTL method were significantly different from those observed using the STL method. While the STL method showed typical features of torsional failure, those that were published in many of previous literatures, including circular abrasion marks and fibrous dimples in the center of this circle, the RTL method showed catastrophic aspects with ruptured surfaces at the cross section as well as the lateral area. Furthermore, while the STL method showed extensive areas of unwinding of the flute at the fracture area, the unwound flutes were rare in the specimens from RTL method. It could be understood that the repetition of a small amount of torsional load at the moment of fracture imposed stress on some of the microcracks without unwinding the flute, but the accumulation of micro failures suddenly resulted in ruptured fractures. Thus, in clinic, the repetitive usage of the NiTi instruments after sterilization may result in a sudden failure without any signs that were found in the STL method such as unwinding.

Since fracture of an instrument in use can potentially lower the probability of healing [18], increasing the resistance to fracture has been a focus in the design of NiTi rotary systems. Because the fracture resistance is strongly correlated with clinical usage, the fracture test should be conducted under conditions that mimic the clinical setting.

Manufacturers of various brands of NiTi rotary instruments have recommended the use of a certain value of torque that is considered safe, that is, capable of preventing (shear) fracture of the instrument. That is not completely accurate because, in the clinical setting, the handpiece (motor) measures the torsional load from the full length of instrument during movement, while the experimental test measures the torsional load at a specific level (3 mm). Thus, in the present study, since the load was concentrated at the 3 mm level of the instruments, the maximum limit of torque was set to 0.5 N·cm.

In present study, constant torsional stress was applied repetitively to simulate repeated locking or autoreverse motion of the rotary file during canal preparation. A higher number of loading cycles to failure implicated a higher resistance to torsional failure of the instrument.

## 5. Conclusions

Under the condition of this study, evaluation of torsional resistance using the RTL method yields similar results to those using the STL method based on ISO specifications. However, RTL method produced different topographic features from STL method. Clinically accumulated torsional stresses may produce different topographic features on the instruments. Testing the torsional resistance of the instruments using the repetitive loading method would be another standard test method for rotary instruments.

## Figures and Tables

**Table 1:** Comparison of torsional resistance (mean ± SD) by the two methods.

Test method	RTL method	STL method
File	NRCF	Ultimate strength (N·cm)
V-Taper 2	5454 ± 1790^a^	1.70 ± 0.19^a^
HyFlex EDM	2373 ± 820^b^	1.35 ± 0.16^b^
ProTaper Next	855 ± 237^c^	0.83 ± 0.05^c^

^a, b, c^Different superscript letters indicate significant differences between groups in vertical column (*p* < 0.05). RTL: repetitive torsional loading; STL: single continuous torsional loading; NRCF: number of repetitive load cycles until fracture.

## References

[1] Cheung G. S. P., Liu C. S. Y. (2009). A retrospective study of endodontic treatment outcome between nickel-titanium rotary and stainless steel hand filing techniques. *Journal of Endodontics*.

[2] Cheung G. S. P. (2007). Instrument fracture: mechanisms, removal of fragments, and clinical outcomes. *Endodontic Topics*.

[3] Yum J., Cheung G. S.-P., Park J.-K., Hur B., Kim H.-C. (2011). Torsional strength and toughness of nickel-titanium rotary files. *Journal of Endodontics*.

[4] Goo H.-J., Kwak S. W., Ha J.-H., Pedullà E., Kim H.-C. (2017). Mechanical Properties of Various Heat-treated Nickel-Titanium Rotary Instruments. *Journal of Endodontics*.

[5] International Organization for Standardization, ISO 3630-1. Dental Root-Canal Instruments: Files, Reamers, Barbed Broaches, Rasps, Paste Carriers, Explorers and Cotton Broaches. Geneve, Switzerland, 1992

[6] Best S., Watson P., Pilliar R., Kulkarni G. G. K., Yared G. (2004). Torsional fatigue and endurance limit of a size 30 .06 ProFile rotary instrument. *International Endodontic Journal*.

[7] Peters O. A., Peters C. I., Schönenberger K., Barbakow F. (2003). ProTaper rotary root canal preparation: Assessment of torque and force in relation to canal anatomy. *International Endodontic Journal*.

[8] Chang S. W., Shim K. S., Kim Y. C. (2016). Cyclic fatigue resistance, torsional resistance, and metallurgical characteristics of V taper 2 and V taper 2H rotary NiTi files. *Scanning*.

[9] ANSI/ADA Specification No. 28, Root canal files and reamers, type K. American Dental Association, Chicago, USA, 2008

[10] Bahia M. G. A., Melo M. C. C., Buono V. T. L. (2006). Influence of simulated clinical use on the torsional behavior of nickel-titanium rotary endodontic instruments. *Oral Surgery, Oral Medicine, Oral Pathology, Oral Radiology, and Endodontology*.

[11] Blum J.-Y., Machtou P., Micallef J.-P. (1999). Location of contact areas on rotary Profile instruments in relationship to the forces developed during mechanical preparation on extracted teeth. *International Endodontic Journal*.

[12] Elnaghy A. M., Elsaka S. E. (2016). Mechanical properties of ProTaper Gold nickel-titanium rotary instruments. *International Endodontic Journal*.

[13] Pedullà E., Lo Savio F., Boninelli S. (2016). Torsional and Cyclic Fatigue Resistance of a New Nickel-Titanium Instrument Manufactured by Electrical Discharge Machining. *Journal of Endodontics*.

[14] Pirani C., Iacono F., Generali L. (2016). HyFlex EDM: Superficial features, metallurgical analysis and fatigue resistance of innovative electro discharge machined NiTi rotary instruments. *International Endodontic Journal*.

[15] Ha J.-H., Kim S. K., Cheung G. S.-P., Jeong S. H., Bae Y. C., Kim H.-C. (2015). Effect of alloy type on the life-time of torsion-preloaded nickel-titanium endodontic instruments. *Scanning*.

[16] Miyazaki S., Imai T., Igo Y., Otsuka K. (1986). Effect of cyclic deformation on the pseudoelasticity characteristics of ti-ni alloys. *Metallurgical Transactions*.

[17] Kaplan W. D. (2015). The mechanism of crystal deformation : Microscopy provides an atomistic view of how crystalline materials deform. *Science*.

[18] Di Fiore P. M., Genov K. A., Komaroff E., Li Y., Lin L. (2006). Nickel-titanium rotary instrument fracture: a clinical practice assessment. *International Endodontic Journal*.

